# COVID-19 AND PSYCHOTROPIC DRUG USE IN ASSISTED LIVING RESIDENTS: VARIATION BY WAVE AND SETTING TYPE

**DOI:** 10.1093/geroni/igad104.1631

**Published:** 2023-12-21

**Authors:** Colleen Maxwell, Hana Dampf, Wajd Alkabbani, Cecilia Cotton, Jean-Michael Gamble, David Hogan, Andrea Gruneir, Matthias Hoben

**Affiliations:** University of Waterloo, Waterloo, Ontario, Canada; University of Alberta, Edmonton, Alberta, Canada; University of Waterloo, Waterloo, Ontario, Canada; University of Waterloo, Waterloo, Ontario, Canada; University of Waterloo, Waterloo, Ontario, Canada; Univeresity of Calgary, Calgary, Alberta, Canada; University of Alberta, Edmonton, Alberta, Canada; York University, Toronto, Ontario, Canada

## Abstract

The onset of COVID-19 was associated with significant, albeit modest, increases in the use of psychotropics and opioids in nursing home residents. Little research exists on whether similar trends occurred among older residents of publicly funded assisted living (AL) homes, a growing and poorly investigated setting. We examined the impact of pandemic wave (1 to 4) and setting type (dementia designated spaces [AL-D] vs other [AL-O]) on prevalent antipsychotic, antidepressant, benzodiazepine, opioid and anticonvulsant use in AL residents from Alberta, Canada. Using linked population-based clinical and health administrative databases, we conducted a repeated cross-sectional study of quarterly medication prevalence from January 2018 to December 2021. Log-binomial GEE models estimated prevalence ratios (PR) for 4 waves (vs 2018-19 historical months) and setting (AL-D vs AL-O) and period-setting interactions. On March 1, 2020, there were 2,874 AL-D and 6,611 AL-O residents in our cohorts (mean age 82.4 vs 79.9 years and 93.5% vs 42.6% with dementia, respectively). Antipsychotic prevalence increased during waves 2-4 for both settings but this increase was significantly greater for AL-D than AL-O in later waves (e.g., AL-D: PR 1.21, 95%CI 1.14-1.27; AL-O: 1.12 (1.07-1.17) for March-May 2021 vs 2018-19). For both settings, there was a significant but modest increase in antidepressants but a decrease in benzodiazepines during several waves. No pandemic effect was observed for opioids in either setting. The AL resident and home characteristics associated with these medication trends, concerns about medication risks (particularly for dementia care settings), and consequent health outcomes for residents require further study.

